# Age-Related Changes and Reorganization of Creativity and Intelligence Indices in Schoolchildren and University Students

**DOI:** 10.3390/jintelligence10030052

**Published:** 2022-08-02

**Authors:** Olga Razumnikova, Maxim Bakaev

**Affiliations:** 1 Psychology and Pedagogy Department, Novosibirsk State Technical University, 630073 Novosibirsk, Russia; razumnikova@corp.nstu.ru; 2 Automated Control Systems Department, Novosibirsk State Technical University, 630073 Novosibirsk, Russia

**Keywords:** creativity, fluid intelligence, academic achievement, execute control, psychometric creativity testing

## Abstract

Despite the lasting interest towards the relationship between intelligence and creativity, comparably less attention is paid to its age-related changes. Our paper considers the organization of fluid intelligence and psychometric indicators of creativity and is based on the experimental data obtained for children aged 11 (*n* = 99) and for young adults (*n* = 77). We used two figural and verbal tasks with and without time limit. We found that the age-related differences in creativity are dependent on the context and the type of testing. The young adults were different from the children, having higher indicators of verbal and figurative creativity, except for the originality of the drawings created within the Incomplete Figures test, and having considerably higher test results for fluid intelligence. These age-related differences, together with the discovered closer relationship between the creativity indicators in the young adults group compared to the children, might suggest insufficient contribution of the components of the executive control of information selection (inhibition, shifting, and updating), which had not fully formed in eleven-year-olds. The comparison of the various indicators of creativity suggests that the most complex task for the children was the composition of an original sentence by joining nouns from various semantic categories.

## 1. Introduction

Age-related changes in creativity indicators remain an urgent problem in psychology, particularly due to the increasing importance of recruiting workers that are highly capable of innovative professional activities. Correspondingly, the development of creative abilities is recognized as one of the most important educational competences in the 21st century (Arrowsmith et al. 2021; Donovan et al. 2014; Ritter and Gemünden 2004). It is known that the development and expression of creative abilities is shaped by a complicated interaction of several personal, cognitive and motivational factors (Jia et al. 2019; Karwowski et al. 2021; Mumford et al. 2012), among which the contribution of intelligence is of particular interest.

Many research publications on age-related development of intellectual abilities, in particular of fluid intelligence, suggest its heterogeneity due to significant individual variability in the processes of age-related differentiation of the brain’s cognitive systems (Cahan and Cohen 1989; Johnson 2011; Riediger et al. 2014). For instance, the reported age-related increase in fluid intelligence at the ages of 7 to 19 is mediated by the degree of development of the related mental processes: their speed, short-term memory and executive functions of attention (Cochrane et al. 2019; Fry and Hale 2000; Chang 2004; Mous et al. 2017).

As for age-related changes in creative abilities, the results are mixed, due to the still debated relationship between intelligence and creativity (Benedek et al. 2014; Frith et al. 2021; Gerwig et al. 2021). Another cause is individual trends in the development of the pre-frontal cortex, which performs regulatory functions in executive control and cognitive flexibility for switching strategies in the search of an original idea, as well as in learning creative activities (Kleibeuker et al. 2016). The leading role of shifting and inhibition in age-related creativity development is highlighted by the results of the analysis of executive functions and of fluid and crystallized intelligence (Krumm et al. 2018). The different dynamics in the creativity indicators has been reported from early adolescence (ages 12 to 13) to adulthood (ages 25 to 30), depending of the employed tasks, with an age-related increase in the fluency of ideas, but similar indicators of originality for verbal divergent thinking (Kleibeuker et al. 2013a).

According to the APT (Amusement Park Theoretical) model, the intelligence provides the base for the creativity together with thematic areas with domain-oriented information (Baer and Kaufman 2005). Numerous studies of the relationship between various components of intelligence and creativity, however, report inconsistent findings: from a close relationship to its absence (Ilagan and Patungan 2018; Jauk et al. 2013; Karwowski et al. 2021; Nusbaum and Silvia 2011; Preckel et al. 2006; Shi et al. 2017; Silvia 2015).

A recent meta-analysis of such studies suggests that the important mediators of the degree of the positive relationship between intelligence and creativity are the experimental conditions, such as an instruction asking to “provide original answers”, the task type: verbal, figurative, etc., or the methods for assessing the originality (Gerwig et al. 2021). At the same time, variability in the relationship between intelligence and creativity might appear due to the different contribution of the functions of the central executive mechanism: inhibition, switching and updating of information processes (Benedek et al. 2014; Pan and Yu 2016). In the latter, the fluency of the idea generation is largely determined by the processes of inhibition, while their originality is determined by intelligence (Benedek et al. 2011). Accordingly, one should expect changes in the relationship between creativity and intelligence, as the effectiveness of executive control of cognitive processes increases in children with age (Razumnikova and Nikolaeva 2019). The effectiveness is commonly considered to be the basis for the children’s successful education (Ribner et al. 2017; Sánchez-Pérez et al. 2018) and a predictor of their intelligence and health (Moffitt et al. 2011).

The development of the executive control leads to the increase in information selection speed and in the effectiveness of the attention focusing on relevant information. The latter is reflected as preferences for analytical processes in searching for remote verbal associations (Wegbreit et al. 2012) or as increase in intelligence scores (Rammsayer and Brandler 2007). Thus, strengthening the inhibitory functions of controlling the release of relevant ideas can lead a decrease in the fluency of answers with an increase in their originality. 

On the other hand, there are data suggesting a positive relationship between flexibility and originality of ideas in creativity testing and weakening of the inhibitory processes that form defocused attention (Carson 2014; Zabelina et al. 2016). The strengthening of the executive control and critical thinking with age can be accompanied by the diminishing role of defocused attention in solving problems. Such opposite dynamics of the processes that underlay creative thinking lead to uncertainty in predicting its performance. To weigh the contribution of different strategies of convergent and divergent thinking in assessing creativity, it was proposed to analyze the ratio of indicators of fluency, flexibility and originality of ideas in different methods for testing creativity (Jia et al. 2019). The diversity in the methods of information processing, training and decision-making strategies based on self-assessment of the originality of the selected answers justifies the need to continue the search for different components in the metacognitive monitoring and the control of creative thinking.

According to the “dual model of creativity,” generation and evaluation of an idea involves switching between the different possible options, rejecting the deviant ones and developing more suitable ones (Kleinmintz et al. 2019). These processes of searching for ideas and selecting them are context-dependent, i.e., they depend on the conditions of the experiment, which can be represented by the parameters of the conceptual space and time of the thinking (Corazza and Lubart 2021). As noted above, the information selection time decreases with age, while the conceptual space increases with age, due to learning (e.g., Dawson-Tunik 2006; Smortchkova and Shea 2020). Consequently, these processes should contribute to increased fluency in the search for ideas and to their diversity and, with successful executive control of the task, to a greater likelihood of selecting an original answer by older children.

There is evidence of a positive relationship between academic performance and creativity (Kim 2020; Palaniappan 2008; Yaghoob et al. 2014). In some other studies, however, it was not found (Atwood and Pretz 2016; Balgiu and Adîr 2014; Olatoye and Akintunde 2010). There are also plenty of findings suggesting a positive relationship between intelligence and academic performance, but the relationship’s strength can be very different, depending on the particular component of intelligence being analyzed and the sample in the study (Behroozi et al. 2011; Erostarbe-Pérez et al. 2022; Morosanova et al. 2015; Nesayan et al. 2019). There are even data that suggest a lack of significant relationship between intelligence and schoolchildren’s academic performance, with a stronger effect of education and the family’s socio-economic status (Alves et al. 2017).

The factors causing variability in the relationships between academic performance and creativity and/or intelligence might include such psychological characteristics of the subjects as degree of executive control and self-regulation of behavior, which are required both in the experimental tasks and the education process (Ishmuratova et al. 2021; Morosanova et al. 2015; Nesayan et al. 2019; Shilshtein and Margalit 2019). On the other hand, the variability can be attributed to the particulars of the experimental methodology, e.g., if there is a time limit, whether the tasks are of verbal or figurative type, if the instructions ask the subjects to provide the most appropriate answer or as many answers as possible (Cropley 1993; Giménez et al. 2019; Hattie 1977; Kaufman and Sternberg 2021).

Thus, the goal of our study was to clarify age-related changes in the relationship between indicators of intelligence and of creativity obtained in different experimental conditions (i.e., with and without time limits) for children and young adults. An additional objective of the study was to determine the relationship between success of learning, intelligence and creativity, since there are different points of view on this issue (Morosanova et al. 2015; Sternberg and Grigorenko 2004; Yaghoob et al. 2014). Some researchers have noted a positive relationship of these variables in younger schoolchildren, diminishing after the age of 9 and disappearing after the age of 17 (Langer et al. 1984). However, some reported a lack of relationships between age and academic performance (DeMeis and Stearns 1992), or even a negative relationship at all grade levels (Grissom 2004).

Considering that only the improvement of executive control of information selection is seen as a stable age-related effect that can influence the relationship between intelligence, creativity and academic performance, we study the relationship of these variables in the groups of participants aged 12 and 19, using the creativity testing conditions that facilitate different types of control in solving the proposed tasks. We believe that such a study is significant because it addresses the relationship between convergent and divergent thinking depending on the content and context of the creative problems.

## 2. Materials and Methods

### 2.1. Participants and Procedure

In our study, there were 99 schoolchildren from the 5th and 6th grades (age 11.7 ± 0.7, 53 boys and 46 girls), further referred to as Gr_1, and 77 university students (age 18.7 ± 1.1, 20 young men and 57 young women), freshmen of the Faculty of Humanities, psychology major, further referred to as Gr_2.

The schoolchildren were from a regular school, whose graduates successfully pass the exams and enroll into universities. The students themselves were rather average graduates of similar schools, since the entry score for the Faculty of Humanities is mediocre. The lack of high intelligence selection bias is supported by the results of the IQ comparison that considers its age norms—the IQ score is not considerably different, being in the range of 87–118 (average 102).

The testing of the schoolchildren was performed in school classes, during two 45-min lessons with a break between them. The testing of the students was conducted in a university auditorium, during a standard 90-min lesson.

### 2.2. Measures

#### 2.2.1. Creativity Tests

To score creativity, a battery of the following tests was used: Recurring Figures (“Circles”) (RF), the Alternate Uses Test (AUT), Incomplete Figures (IF) and the composition of original sentences (SCT) using five triads of words—nouns referring to different semantic categories. The tests formats (type and context) had 2 levels: verbal–figurative, with–without time limits. Correspondingly, the tests were: verbal with a time limit (AUT), verbal without a time limit (SCT), figural with a time limit (RF) and figural without a time limit (IF).

The time allocated to complete each of the first two tasks was limited to 5 min, while IF and SCT had no time limitation and actually took about 20 min. The instructions for completing each task would say: “try to come up with the most original answer: a drawing, a way of using the object (e.g., newspaper), or a meaningful sentence”. To understand which answer should be considered an original one, some examples of original and typical answers were provided for each task, as we describe below.

**Figure 1:**
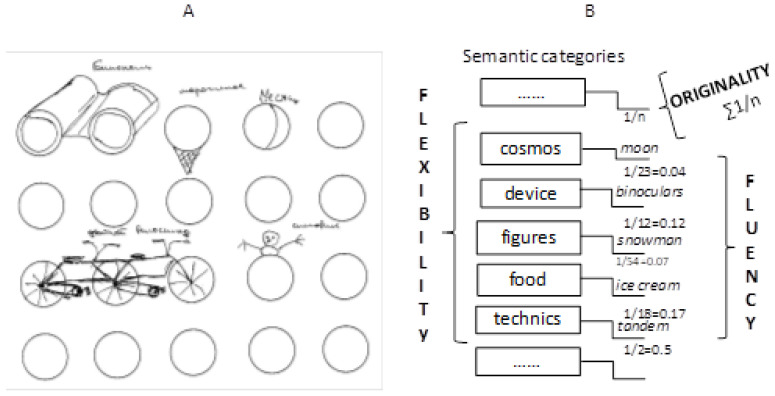
(**A**) An example of a fulfilled RF test; (**B**) The creativity indicator calculation scheme in the software for the same example.

To quantify the indicators of creativity (originality, fluency and flexibility), computerized methods were used. Originality was calculated as the reciprocal of the number of the same answers recorded in the database, flexibility was calculated as a variety of semantic categories to which the answers belonged, and fluency was calculated as the number of answers provided for the test (Razumnikova 2002). An example of fulfilling the RF test and the calculation of the related creativity indicators, together with screenshot demonstrating the computerized version of the test, is provided in Figure 1. In the example, the indicators of originality, fluency and flexibility were 1.25, 5 and 5, respectively. The assessment of the creativity indicators in AUT and IF tests was conducted using a similar software. Its database contained the answers collected when testing the university students (220 < *n* < 248 for the three above mentioned tests). The expert assessment of the originality of the sentences in the SCT was based on the following system: 0—stereotyped idea, 2—original idea, 1—intermediate result; with the summing up of the points for the composed sentences. For instance, for the three words “tornado”, “computer” and “pin”, the sentence “the sudden tornado took away the computer and the pin” would get 0 points, whereas the sentence “the news that the Wizard’s pins had turned Scarecrow’s brain into a computer has spread throughout the Emerald City like a tornado” would get 2 points.

The Sentence Completion Test was scored by 3 raters, and the intra-rate reliability was 0.80. In the three other tests, one rater would score creativity, as we previously found that in the computerized version of the test, the intra-rate reliability for several raters would amount to 0.88–0.92. The answers were encrypted, and the rating process was blind in regard to both the responses and the participants.

#### 2.2.2. Intelligence Tests

The 60-item version of Raven’s Standard Progressive Matrices was used as a measure of general fluid intelligence (IQf). The limit of the time allocated for the testing was 20 min. The number of correct answers was analyzed in each session.

## 3. Results

### 3.1. Creativity and Intelligence in the Two Age Groups

According to the descriptive statistics, the indicators of creativity, except for the IF test, had left-side asymmetry in both groups, suggesting a high number of stereotyped answers. The creativity and intelligence scores for Gr_1 and Gr_2, as well as the between-group comparisons with Mann–Whitney statistics are presented in Table 1.

The results suggest that Gr_2 has demonstrated higher scores of both creativity and intelligence compared to Gr_1 in all tests, except for IF test, in which fluency and originality did not differ significantly between the groups, and only flexibility was higher in Gr_2 than in Gr_1.

**Figure 2:**
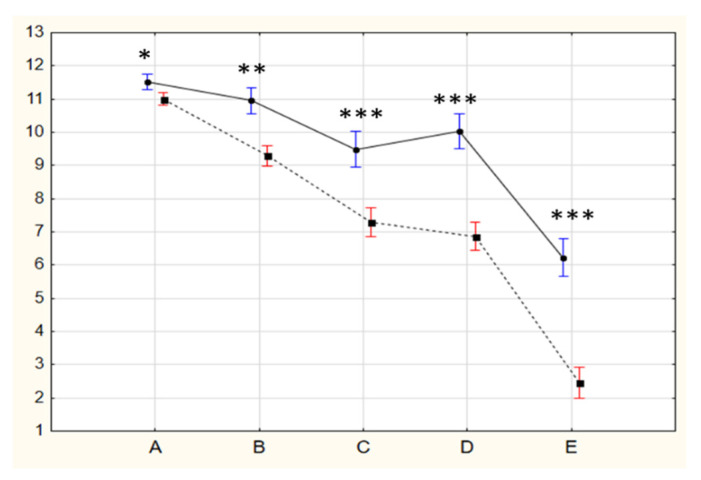
The numbers of correct answers in the five series of the Raven’s test for schoolchildren (dashed line) and students (solid line). Significance is *—*p* < 0.0002, **—*p* < 0.00005, ***—*p* < 0.000001.

The results for the five series in Raven’s test are presented in Figure 2. According to the between-group comparisons, all the scores in Gr_2 were higher than in Gr_1 (Mann–Whitney *p* < 0.0002). Figure 2 demonstrates that the age-related differences in the test performance increase together with the increasing sophistication from series A to series E.

The results of the correlation analysis of the data performed to explore the relationship between the creativity indicators are presented in Table 2 and Table 3. The tables present the values of significant Spearman correlation coefficients (0.00001 < *p* < 0.05) or “n/s” for the non-significant coefficients. The acronyms for the indicators in our subsequent analysis of the data are as follows:

- FlRF, FxRF and OrRF are indicators of fluency, flexibility and originality in figurative tests with recurring figures;
- FlIF, FxIF and OrIF are indicators of fluency, flexibility and originality in figurative tests with incomplete figures;
- FlAUT and OrAUT are indicators of fluency and originality in verbal tests for alternate uses;
- FlSCT and OrSCT are indicators of fluency and originality in verbal tests for sentence completion.

The results suggest that in Gr_2 the creativity indicators were more tightly related than in Gr_1 (20 significant correlations in Gr_2 vs. 12 significant correlations in Gr_1). We would like to especially note OrSCT, which had no significant correlations with any of the other creativity indicators in Gr_1, whereas in Gr_2 OrSCT was positively correlated with OrAUT, OrIF and FlSCT.

As for the relationship between creativity and intelligence, only in Gr_2 did we find the tendency towards the positive relationship of IQf (the total number of correctly performed tasks in the A–E series) with OrIF and FlSCT (0.06 < *p* < 0.09).

### 3.2. The Effect of Differences in the Academic Performance on the Creativity and Intelligence Organization

Our next research task was the exploration of the relationship between academic performance and the indicators reflecting the organization of creativity and intelligence. For the schoolchildren, the academic performance score was their averaged grades for each subject in the first two quarters of the study year. For the students, the academic performance score was their averaged grades for each subject in the first semester (half-year). To that end, in both groups we identified sub-groups based on the academic scores. The sub-group Gr_1_0 included the schoolchildren who had their average academic score lower than 4 out of 5 (*n* = 36), whereas Gr_1_1 included the schoolchildren with the score of higher than 4 (*n* = 63). The groups Gr_2_0 (*n* = 41) and Gr_2_1 (*n* = 36) were formed in a similar manner for the students.

The comparison of the sub-groups differing in academic scores has revealed a significant difference only for OrIF in Gr_2, with higher values of the indicator in Gr_2_1 than in Gr_2_0 (*p* < 0.04). There was also the trend towards higher values of FlSCT in Gr_2_1 compared to Gr_2_0 (*p* < 0.1), as well as of FxRF and FlAUT in Gr_1_1 compared to Gr_1_0 (*p* < 0.1).

**Figure 3:**
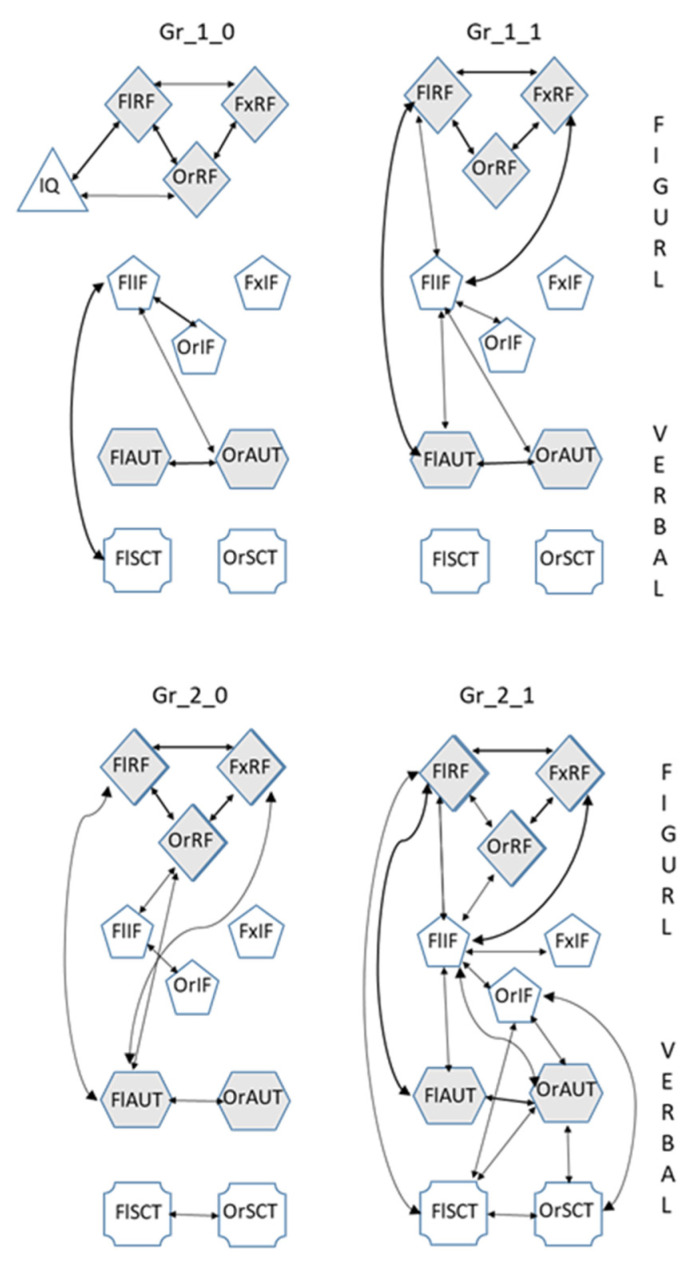
The relationships between the indicators of creativity due to age and academic scores. Gr_1_0 or Gr_1_1 and Gr_2_0 or Gr_2_1 are the sub-groups of schoolchildren and students, respectively, with lower or higher average academic score. Thicker lines with arrows show correlations significant at 0.01. Thinner lines show correlations significant at 0.05.

The results of the correlations analysis for the indicators of creativity and intelligence for all the four sub-groups are presented in Figure 3. It demonstrates not only the age-related increase in the relationships of the indicators, as Table 2 and Table 3 do, but also the effect of the academic scores.

Gr_1_0 had less pronounced relationships between the different indicators of verbal and figural creativity compared to Gr_1_1. Also, only Gr_1_0 had significant relationships with IQf. Gr_2_1 had the tightest relationships between the various indicators of creativity compared to other groups, particularly for the indicators of verbal creativity FlSCT and OrSCT, whose relationship was significant in Gr_2_1 only (see Figure 2).

### 3.3. The Effect of the Different Types of Creativity Tests and the Time Limit

Furthermore, we sought to identify the particulars of the relationships between the indicators of creativity due to age, also considering the nature of the tests: verbal or figural, with or without time limit. To this end, we normalized all the indicators and used MANOVA with the independent variables AGE (2) and GROUP (2) and the dependent variable CREATIVITY (10).

**Figure 4:**
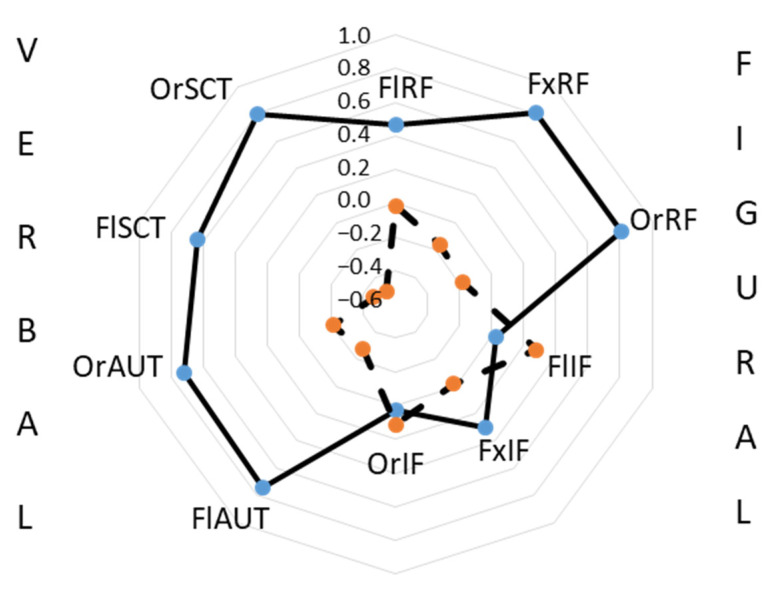
The normalized indicators of creativity in the group of schoolchildren (dashed line) and in the group of students (solid line).

In addition to the between-group differences due to AGE and GROUP described above (correspondingly, F_1,123_ = 48.9, *p* < 0.000001, η^2^ = 0.29; F_1,123_ = 6.27, *p* < 0.000001, η^2^ = 0.05), we found a significant interaction between AGE and CREATIVITY (F_9,1107_ = 12.2, *p* < 0.0000001, η^2^ = 0.09). Post-hoc analysis with the Bonferroni correction has confirmed the significance of the between-group differences (see in Table 1) and found differences between all the variables (*p* < 0.001), except for FlIF and OrIF (see Figure 4). The planned comparison found that in Gr_2, the normalized OrRF was higher than FlRF, and OrRF was higher than OrIF, while in Gr_1, OrRF was lower than OrIF (0.00006 < *p* < 0.001). The relationships between FlIF and FxIF were inconsistent, as in Gr_1 FlIF was higher than FxIF, while in Gr_2 FlIF was lower (*p* < 0.000001). The profile of the originality indicators in Gr_1 was dominated by OrIF, whereas in Gr_2 this indicator was the lowest compared to all the others (Figure 4).

## 4. Discussion

The available literature is somehow contradictory with respect to the relationship between age, intelligence, creativity and academic performance. The common ground is that executive control improves with age in the period of 12 to 17 years old. Therefore, we compared the relationship between intelligence, creativity and academic performance in two purposefully different age groups that are known to be different in the development of executive control. We sought to explore its role in various conditions of creativity testing (verbal or figural, with or without a time limit), since these conditions require different degrees of the executive control of information selection.

Higher values of both intelligence and creativity indicators in Gr_2 in all the tests, except for IF, can be considered as the evidence of age-related acceleration in information processing in the two considered age groups. This finding is consistent with the other data from the analysis of intelligence or verbal creativity (Frischkorn and von Bastian 2021; Rammsayer and Brandler 2007; Wegbreit et al. 2012). The trend found in Gr_2 towards a positive relationship between intelligence and individual indicators of creativity (OrIF and FlSCT) suggests the contribution of intelligence only when performing tasks that require the rejection of a stereotypical response to stimuli under conditions of their single presentation. In this case, the importance of effective processes of inhibition of the first response that pops up from the memory and switching to the search for another option increases. This happens because finding an original solution to the problem requires abandoning close stable associations and transition to searching for an idea in distant semantic structures. Indeed, it was found that young adults with low creativity are characterized by more rigid semantic memory structure compared to the ones with high creativity (Kenett et al. 2014).

On the other hand, it has been shown that ten-year-old children with high indicators of divergent thinking (assessed using the AUT test) had a flexible structure of the semantic network, while the ones with high fluid intelligence (IQf) were characterized by its more rigid organization (Rastelli et al. 2020). Apparently, the age-related effect of increased rigidity in the organization of the semantic network as a result of training may be the reason that, in Gr_1, there was not a single sentence that had an original meaning. For the most part, having completed one sentence according to the stereotypical grammatical rules, the schoolchildren would stop searching for further options. Whereas the combination of a decrease in the information selection time with an increase in the conceptual space with age ( Dawson-Tunik 2006; Smortchkova and Shea 2020) provided higher rates of both FlSCT and OrSCT in Gr_2.

Our results differ from the data obtained when comparing similar participants in age, but small groups (22 people in each group) of schoolchildren aged 10–12 and university students from Hong Kong aged 19–22 performed the verbal, figural and real-problem tasks (Wu et al. 2005). The authors explained the absence of between-group differences in verbal creativity by the dominance of students when solving a real-problem task, and schoolchildren when performing a figural task, by an interaction between task structures and knowledge bases that increase with age. However, according to the results of another study, on the contrary, an increase in visual-spatial divergent thinking fluency and a decrease in OrAUT were found in the 18–19 age group compared to children aged 12–13 (Kleibeuker et al. 2013a). Brain activity analysis using fMRI has revealed the activation of the parietal and temporal regions of the left hemisphere during AUT and additional involvement of the prefrontal cortex, associated with more successful generation of creative ideas in young adults than in adolescents aged 15–17 (Kleibeuker et al. 2013b). The authors explained these differences with the yet insufficient optimization of the processes of generating ideas and their selection in adolescence.

Our data can be interpreted according to this hypothesis too. Moreover, such an effect of insufficient optimization of the generation of ideas in Gr_1 is weaker than in the others in IF, that is, when creating drawings based on a variety of figurative stimuli without a limit on the thinking time, which reduces the requirements for both the speed of generation of ideas and their critical selection. Under these conditions, the age differences concern FxIF only—apparently, as a consequence of yet insufficient development of the conceptual space in childhood. Thus, different trajectories of the formation of inhibition processes, semantic networks and cognitive flexibility can be the reason for the inconsistent results in the relationship between the indicators of creativity and intelligence and the age-related dynamics noted above.

It is noteworthy that when comparing the sub-groups differing in academic scores, higher OrIF values were noted in Gr_2_1 than in Gr_2_0. Taking into account that at the same time, the tendency towards higher indicators of creativity, FlSCT in Gr_2_1 than Gr_2_0, as well as FxRF and FlAUT in Gr_1_1 than in Gr_1_0, it can be assumed that in the absence of between-group differences in IQf, the discovered effects reflect the particulars of variable combinations of knowledge bases and the flexibility of their application that improve with age and with learning.

To explain the different forms of the relationship between intelligence and creativity, the Space-Time Continuum (Corazza and Lubart 2021) could be used. According to this approach, the intelligence construct is dominant in a condition of tight space and tight time, whereas the creativity construct is dominant in loose space and loose time, and these constructs can overlap more or less depending on the context characteristics. Therefore, the presence of a positive relationship between IQf and FlRF/OrRF only in Gr_1_0 can be explained by the combination of tight space and tight time during the RF testing.

The different conditions in our experiment for the creativity testing, i.e., with time limit and without it, result in four possible idea-seeking options in the Space-Time Continuum, among which are the loose-space/loose-time quadrant (in this quadrant the creativity construct is dominant (Corazza and Lubart 2021)), representing relatively smaller age-related differences, whereas the limitations in time or in conceptual space strengthen them.

According to the results of the correlation analysis, Gr_1_0 differed from all others in the minimal coherence of creativity indicators, indicating the spontaneity of responses due to the weak co-organization of different components of creative thinking. This effect was especially noticeable when comparing Gr_1_0 with Gr_2_1, in which the indicators of creativity were correlated the most closely (see Figure 3).

Previously, when analyzing the EEG correlates of creativity using the same testing conditions, we have demonstrated the differences in the functional activity of the cerebral cortex depending on the psychometric indicators of originality (Razumnikova 2021). The considerable synchronization of biopotentials found at the alpha frequency in the anterior part of the cortex for highly creative individuals (young adults), compared to the low-creative ones, is considered as a “pre-adjustment” of the background state of the brain (Kounios et al. 2008) that leads to the subsequent successful processes of internal attention and inhibition of irrelevant information in divergent thinking (Benedek et al. 2011). The regional specificity of such “pre-tuning” of the brain was that verbal originality was reflected in the activity of the temporal and central-parietal areas of the cortex, and figurative originality was reflected in the parietal-occipital areas (Razumnikova 2021). It is known that the resting-state activity of the brain presents frontal and more posterior brain regions appear to underlie both intelligence and creativity (Frith et al. 2021) and allows differentiating intellectually gifted children (Liu et al. 2008). Therefore, along with the development of the executive functions of the nervous system, one should expect that with age individually more stable strategies for solving problems are formed, combining convergent and divergent thinking depending on the content and the context of the problems. Indeed, the analysis of age-related particulars of the relationship between intelligence and creativity has shown it to be more stable in older children (Nikolaeva et al. 2018).

The common factors affecting the efficiency of cognitive processes are their speed and the amount of the working memory required, which is determined by the complexity of the task (Goecke et al. 2021). With the same time conditions for testing the intellectual abilities in Gr_1 and Gr_2, the discovered between-group differences in the effectiveness of solving tasks of different series demonstrate the effect of complexity: no differences in series A and increasingly significant dominance of Gr_2 in series D and E (see Figure 2).

When comparing the indicators of creativity, we can conclude that verbal creative tasks are more difficult for children than figurative ones. The figurative RF task, in turn, is more difficult when compared to IF, due to the repetition of stimuli under time-constrained conditions. In this case, the lower fluency of ideas in Gr_1 compared to the one in Gr_2 can be related to slower image retrieval from the memory. The lower FxRF can be related to more limited semantic resources, which accordingly causes the low OrRF values. In the context of IF testing, these factors become non-critical, so that the children can generate images that do not differ in originality from Gr_2.

The tight relationship of all the creativity indicators in Gr_2 might indicate the existence of a general decision-making mechanism under the influence of executive control, including multiple numerations of options and giving a more meaningful weight to one idea of an answer and ignoring the other, due to experience-based understanding of the differences between stereotyped and original answers. In Gr_1, and especially in Gr_1_0, all of the above components of executive control were less developed, which increased the likelihood of stereotyped decisions being extracted. Therefore, it is especially difficult for children to cope with the solution of verbal problems, since they rely on the initial meaning of the utterance, which had been strengthened in the process of mastering speech and the subsequent schooling.

## 5. Limitations and Further Research

We did not consider the effect of gender on age-related changes in the relationship between creativity and intelligence. Some studies report that relationships between creativity, intelligence and academic performance do not differ significantly in younger schoolchildren (Pastor and David 2017) or undergraduate students (Naderi et al. 2010) due to gender. However, given that executive control is formed in girls earlier than in boys, it could be advisable to take this factor into account. We have considered only the ratio of fluid intelligence and verbal and figural creativity, which were measured by the four tasks. It is necessary to further analyze the influence of different models of intelligence, including the influence of the verbal and visual-spatial components of intelligence and age-related changes in creativity, taking into account the nature and the context of its testing.

Another limitation of our study is the sampling: we have selected our subjects only from one regular school for the schoolchildren and one faculty for university students. The two groups in our study significantly differed in age, but had relatively small age variability within groups, so we did not rely on regression analysis. Our goal was not to explore the effect of age on each of the indicators, but to study them in interaction: intelligence, creativity and academic performance in the age groups that differ in executive control of behavior.

The measurement of originality as relative infrequency might be problematic, and in our study it was supplemented by the Consensual Assessment Technique by the experts. However, we did not consider the agreement between the two measurements.

We relied on the existing literature to explain the discovered age-related particulars in the relationship between creativity, intelligence and academic scores by the differences in the formation of executive control of behavior. It could be useful to consider the importance of the executive control using experimental conditions to determine it.

Additionally, personality and motivation characteristics associated with both age and gender might be the mediators that significantly influence the relationships between creativity, intelligence, executive control and academic performance. In addition, we did not attempt to isolate the factors of education and age (unlike, e.g., in Cahan and Cohen 1989), as both the schoolchildren and the students were sampled to be representative of the general population, i.e., having a rather typical education level for their age group.

## 6. Conclusions

Age-related differences in creativity depend on the context and the type of testing and are weakened under the conditions of presentation of different imaginative stimuli without limiting the thinking time. The young adults differed from the eleven-year-old children in higher indicators of verbal and imaginative creativity, with the exception of the originality of the drawings created using the Incomplete Figures test, as well as in significantly better results of the fluid intelligence testing. These age differences, together with the discovered tighter relationship of creativity indicators in the group of young adults as compared to the group of children, may indicate an insufficient contribution of the components of the executive control of information selection (inhibition, shifting and updating), which are not yet fully developed in eleven-year-old schoolchildren, in solving the tasks. The comparison of the different creativity indicators suggests that the most difficult task for the children was to compose a sentence that has an original meaning and to combine nouns from different semantic categories.

## Figures and Tables

**Table 1:** The creativity indicators for the schoolchildren and the university students and their comparisons.

Variable	Gr_1 (Schoolchildren)					Gr_2 (University Students)					*p* ^1^
	Mean	SD	Med	Min	Max	Mean	SD	Med	Min	Max	
Recurring figures (RF)											
Fluency	10.8	4.9	11.0	2.0	20.0	14.0	6.0	15.0	3.0	25.0	0.001
Flexibility	5.3	2.4	5.0	1.0	13.0	8.4	3.6	9.0	2.0	16.0	0.000
Originality	0.7	0.7	0.6	0.01	3.4	1.6	1.4	1.2	0.1	5.6	0.000
Alternate Uses Test AUT)											
Fluency	6.2	2.7	6.0	1.0	12.0	9.7	3.5	9.0	4.0	18.0	0.000
Originality	1.0	1.0	0.5	0.01	4.6	2.0	1.5	1.8	0.2	6.6	0.000
Incomplete Figures (IF)											
Fluency	9.7	1.1	10.0	3.0	10.0	9.6	1.0	10.0	5.0	10.0	n/s
Flexibility	5.8	1.4	6.0	2.0	9.0	6.4	1.3	7.0	3.0	8.0	0.005
Originality	3.1	1.3	3.0	0.5	6.9	3.4	1.6	3.4	0.2	7.3	n/s
Sentence Completion Test (SCT)											
Fluency	5.0	1.5	5.0	0.0	13.0	8.7	3.7	8.0	5.0	17.0	0.000
Originality	1.0	1.3	0.0	0.0	5.0	5.3	3.9	4.0	0.0	18.0	0.000
Raven’s Test (IQf)											
Total score	34.5	8.6	36.0	7.0	50.0	48.2	5.7	49.0	34.0	58.0	0.000

^1^ n/s is non-significant.

**Table 2:** The correlation coefficients for the creativity indicators for the schoolchildren.

Indicator	FxRF	OrRF	FlAUT	OrAUT	FlIF	OrIF	FlSCT
FlRF	0.51 ***	0.59 ***	0.34 *	n/s	n/s	n/s	n/s
FxRF		0.43 **	0.26 *	n/s	0.34 *	n/s	n/s
OrRF			n/s	n/s	0.26 *	n/s	n/s
FlAUT				0.68 ***	0.28 *	n/s	n/s
OrAUT					0.30 *	n/s	n/s
FlIF						0.32 *	0.32 *

Significance is *—*p* < 0.005, **—*p* < 0.001, ***—*p* < 0.00001, or n/s—non-significant.

**Table 3:** The correlation coefficients for the creativity indicators for the students.

Indicator	FxRF	OrRF	FlAUT	OrAUT	FlIF	FxIF	OrIF	FlSCT	OrSCT
FlRF	0.76 ***	0.72 ***	0.54 ***	n/s	0.37 *	n/s	n/s	0.39 *	n/s
FxRF		0.57 ***	0.44 **	0.33 *	n/s	n/s	n/s	n/s	n/s
OrRF			0.44 **	0.30 *	0.37 *	n/s	0.29 *	n/s	n/s
FlAUT				0.36 *	0.31 *	n/s	n/s	n/s	n/s
OrAUT					n/s	n/s	n/s	n/s	0.35 *
FlIF						0.31 *	0.37 *	n/s	n/s
FxIF							n/s	n/s	n/s
OrIF								0.38 *	0.39 *
FlSCT									0.38 *

Significance is *—*p* < 0.05, **—*p* < 0.005, ***—*p* < 0.00001, or n/s—non-significant.

## Data Availability

The data presented in this study are available on request from O.R. (the first author). The data are not publicly available due to privacy reasons.
